# Ductal Intervention in Chronic Pancreatitis: Impact on Glycemic Control and Endocrine Insufficiency Management

**DOI:** 10.7759/cureus.66378

**Published:** 2024-08-07

**Authors:** Sidharth Harindranath, Biswa R Patra, Abu A Ansari, Arun Vaidya, Ankita Singh, Sridhar Sundaram, Aniruddha Phadke, Akash Shukla

**Affiliations:** 1 Department of Gastroenterology, King Edward Memorial Hospital and Seth Gordhandas Sunderdas Medical College, Mumbai, IND

**Keywords:** pancreas endosopy, steatorrhea, endoscopic retrograde cholangio-pancreatography, diabetes mellitus, pancreatitis chronic

## Abstract

Background and aim

Pancreatic endotherapy has been established as a viable and effective modality for the management of pain in chronic pancreatitis (CP). However, its impact on endocrine insufficiency has been rarely reported. In this retrospective study, we aimed to assess the impact of endotherapy on glycemic status and the management of diabetes in these patients.

Methods

A retrospective review of a prospectively maintained database of patients with CP with pain presenting to the King Edward Memorial Hospital and Seth Gordhandas Sunderdas Medical College, Mumbai, India, from December 2021 to May 2023 was done. Detailed clinical, laboratory, imaging, and treatment data were recorded. Endocrine dysfunction was defined as glycosylated hemoglobin (Hba1C) ≥6.5 g/dl. The status of endocrine function (Hba1C values) before and after endotherapy, as well as the requirement of oral hypoglycemic agent (OHA) and/or insulin, was recorded.

Results

One hundred forty-one patients underwent endoscopic retrograde cholangiopancreatography for the management of pain (mean age: 35 years, 74.5% males). Prior to endotherapy, pathological endocrine dysfunction was seen in 60 patients (42.5%). The mean HbA1c value was 8.46 g/dl (4.5-16.1g/dl). OHAs alone were used in 13/60 (21.6%), and 34/60 (56.6%) required insulin. A combination of OHA and insulin was required in 13/60 (21.6%) of patients. Post-endotherapy, none of the patients were on a combination of OHAs and insulin; 5/13 (38.4%) patients were on OHAs alone, while 8/13 (61.5%) patients were shifted to insulin. Out of the total 47 patients who required insulin, insulin could be stopped in 15/47 (31.9%) of patients. Patients who demonstrated improvement in endocrine dysfunction had significantly lower HbA1c values (6.38 vs. 8.07 g/dl, p < 0.001), a higher proportion of patients with idiopathic pancreatitis (73.3% vs. 22.2%, p = 0.004), and a lower proportion of patients with concomitant exocrine insufficiency (13.3% vs. 53.3%, p = 0.007).

Conclusions

One-third of the patients had improvements in endocrine dysfunction. Early ductal intervention in a selected subset of patients with CP may have the potential to improve glycemic status.

## Introduction

Chronic pancreatitis (CP) is a generally irreversible chronic pathological condition that involves repeated cycles of inflammation, increasing fibrosis, and the final loss of exocrine and endocrine tissue. The parenchyma and pancreatic duct exhibit distinct and recognizable morphological alterations that significantly influence the clinical presentation and treatment plan. Persistent abdominal pain is the most typical clinical presentation, and it greatly lowers quality of life [1]. The multitude of mechanistic pathways in the pathophysiology of pain may explain the partial or no response to treatment in a significant proportion of patients [2].

There are currently few options available that can change the disease’s natural course. Currently available therapeutic options include surgery, endotherapy, dietary and lifestyle modifications, and conservative analgesic medication. The majority of these therapies are directed toward pain management. The management of CP requires a multimodal approach as the pathogenesis involves various dependent and independent mechanisms [3,4].

Endoscopic therapy typically involves endoscopic retrograde cholangiopancreatography (ERCP) with pancreatic sphincterotomy, followed by the extraction of stones with or without the use of extracorporeal shockwave lithotripsy (ESWL), the placement of a pancreatic ductal stent, and/or dilatation of pancreatic duct strictures [5]. Diabetes secondary to CP has been attributed to functional loss of islet cells due to inflammatory cell infiltration into the islets and islet cell fibrosis [6]. Additionally, nutrient maldigestion leads to impaired incretin secretion and decreased insulin release from remaining beta cells [7]. Although endotherapy has demonstrable efficacy in the management of pain, its role in the management of pancreatogenous diabetes is vague. With this background, we aimed to analyze the impact of ductal intervention on the glycemic status and management of endocrine dysfunction in patients with CP.

## Materials and methods

Study setting

This study was carried out after obtaining approval from the institutional ethics committee (IEC-22/2/2023). A waiver of consent was obtained. A retrospective review of a prospectively maintained database of patients with CP presenting to a dedicated pancreas clinic in the Department of Gastroenterology of King Edward Memorial Hospital and Seth Gordhandas Sunderdas Medical College in Mumbai, India, was done. This was carried out from December 2021 until May 2023. The inclusion criteria were reviewed for all patients with CP undergoing ERCP for the management of intractable pain. Clinical history, records of previous imaging, and treatment were recorded in a structured proforma.

Exclusion criteria

Exclusion criteria included patients for whom endotherapy was not feasible, patients who had undergone prior pancreatic surgery, and patients with diagnosed malignancies. Patients without complete or verifiable data or who were lost to follow-up were excluded.

Parameters

Detailed clinical, laboratory, imaging, and treatment data were recorded. The etiology of CP, morphology of the duct, presence of ductal calculi, dominant strictures, side branch dilatation, common bile duct stricture, presence of pseudocyst, splanchnic venous thrombosis, and presence/absence of pancreatic head mass were noted. Details of pre-endoscopic imaging with CT or magnetic resonance cholangiopancreatography (MRCP) were noted. The presence of steatorrhea, glycemic status, and details of endoscopic interventions were noted.

Definitions and diagnoses

The diagnosis of CP was established by fulfilling at least one of the following criteria:

Imaging Findings

The presence of pancreatic calcifications is demonstrable on plain abdominal radiography, transabdominal ultrasound, or CT.

Pancreatography Changes

Moderate-to-marked alterations in the pancreatic duct were observed during pancreatography, as defined by the Cambridge classification system [8].

Endoscopic Ultrasonography

A combination of ductal and parenchymal changes was identified on endoscopic ultrasonography using the Rosemont criteria [9].

Evaluation of Pancreatic and Biliary Strictures

MRCP was used to assess pancreatic and biliary strictures, with confirmation obtained during ERCP. The main pancreatic duct was considered dilated if its maximum diameter on the MRCP exceeded 5 mm. A dominant stricture was defined as per a previously published study [10].

Risk Factor Assessment

Alcohol: Alcohol consumption exceeding 50 grams per day for at least five years was categorized as a risk factor for CP.

Smoking: A smoking history exceeding 10 pack-years was considered a risk factor.

Idiopathic CP: In the absence of identifiable risk factors, the disease was classified as idiopathic.

Diagnosis of diabetes

The American Diabetes Association (ADA) criteria were utilized to diagnose diabetes [11]. Blood sugar measurements for diagnosis were performed outside of periods of acute abdominal pain or documented acute pancreatitis episodes. CP-induced diabetes was defined by meeting at least one of the following criteria: diagnosed subsequent to the onset of CP, developed before the age of 40 in conjunction with CP, and/or associated with low C-peptide levels in the context of CP.

Pancreatic exocrine insufficiency (PEI)

The presence of steatorrhea (fatty stools) alongside significant pancreatic atrophy and/or pancreatic parenchymal and ductal calcifications with calculi was used to diagnose PEI.

Intervention

The patients were treated as per the institute’s standard management protocol. Treatment approaches included antioxidants, non-steroidal anti-inflammatory drugs, pancreatic enzyme supplements with enteric coating, and pregabalin or selective serotonin reuptake inhibitors for suspected pancreatic neuropathic pain. Tramadol hydrochloride was administered for acute pain episodes. Due to their limited availability in India, high-potency opioids like morphine and codeine derivatives were not utilized. Acute pancreatitis episodes were managed conservatively, following standard protocols. Diabetes management began with oral hypoglycemic agents (OHAs), transitioning to insulin therapy under the guidance of an in-house endocrinologist if unresponsive.

Follow-up and response assessment

Patients were followed up at six-month intervals. Data pertaining to pain, other symptoms or complications, treatment details, whether on OHA and/or insulin, the presence of steatorrhea, and compliance with medications were noted. Relevant blood tests (glycosylated hemoglobin (HbA1c)) and abdominal imaging were performed. Improvement in glycemic status was defined according to the ADA guidelines [11] as average HbA1c <6.5 g/dl and/or fasting blood glucose <126 mg/dl. Improvement in steatorrhea was judged subjectively as an improvement in stool consistency and a decrease in stool frequency.

Statistical analysis

Data were expressed as mean ± SD, as a percentage of the total number of patients, or as a median with an interquartile range. Categorical data were expressed as percentages. Continuous data were compared using the Student’s t-test or Mann-Whitney U test as appropriate. Categorical variables between the two groups were compared using the chi-square test.

## Results

One hundred forty-one patients (n = 141; mean age: 35 years; males: 105 (74.5%)) who underwent ERCP for chronic debilitating pain despite optimal analgesia were included in the final analysis. A pancreatic sphincterotomy was done in all patients (100%). Post-ERCP pancreatitis was seen in 10 patients (7.1%), and two patients (1.4%) had a post-sphincterotomy bleed. All complications were successfully managed conservatively. Baseline characteristics and disease morphology of patients are summarized in Table 1.

**Table 1:** Baseline patient characteristics and disease morphology CBD, common bile duct; MPD, main pancreatic duct

Parameter	Value (n = 141)
Age	35 (17-73)
Male gender (n; %)	105 (74.5%)
Etiology	
Alcohol (n; %)	76 (53.9%)
Idiopathic (n; %)	53 (37.5%)
Hereditary (n; %)	7 (5%)
Trauma (n; %)	5 (3.5%)
Steatorrhea (n; %)	56 (39.7%)
Diabetes (n; %)	60 (42.5%)
Disease morphology	
MPD diameter (median; IQR) mm	5.5 (3.4-6.4)
Pancreatic ductal calculi (n; %)	66 (46.8%)
Dominant MPD stricture (n; %)	47 (33.3%)
Intraductal calculi and stricture (n; %)	9 (6.4%)
Parenchymal/side branch dilatation (n; %)	61 (43.3%)
CBD stricture (n; %)	10 (7.1%)
Pseudocyst (n; %)	21 (14.9%)
Splanchnic venous thrombosis (n; %)	13 (9.2%)
Pancreatic head mass (n; %)	13 (9.2%)

Fifty-six patients (39.7%) had steatorrhea subjectively, which improved in two of 56 patients (3.5%). Prior to endotherapy, pathological endocrine dysfunction was seen in 60 patients (42.5%). The mean HbA1c value was 8.46 g/dl (4.5-16.1g/dl). OHAs alone were used in 13/60 (21.6%), and insulin alone was required in 34/60 (56.6%) of patients. A combination of OHA and insulin was required in 13/60 (21.6%) of patients. Post-endotherapy, none of the patients were on a combination of OHAs and insulin; 5/13 (38.4%) patients were on OHAs alone, while 8/13 (61.5%) patients were shifted to insulin. Out of the total 47 patients who required insulin, insulin could be stopped in 15/47 (31.9%) of patients.

On comparison of patients with and without improvement in glycemic parameters post-endotherapy, the idiopathic etiology of CP was significantly associated with improvement in glycemic status, whereas the presence of steatorrhea prior to endotherapy was associated with poor response (53.3% vs. 13%, p = 0.007). The mean HbA1c values were significantly lower in patients who experienced an improvement (6.38 ± 1.64 vs. 8.07 ± 1.84 g/dl, p < 0.001). The mean MPD diameter was greater in patients who demonstrated improvement in glycemic function (6.14 vs. 5.46, p = 0.051). The comparison of patients with and without improvement in endocrine dysfunction post-endotherapy has been summarized in Table 2.

**Table 2:** Comparison of patients with and without improvement in endocrine dysfunction post-endotherapy CBD, common bile duct; Hba1C, glycosylated hemoglobin; MPD, main pancreatic duct

n = 60	Improvement (n = 15)	No improvement (n = 45)	p-value
Age (mean ± SD) years	43.2 ±14.3	46.2 ± 12.3	0.14
Male, n (%)	12 (80%)	30 (66.6%)	0.651
Etiology: idiopathic alcoholic	11 (73.3%); 4 (26.6%)	10 (22.2%); 29 (64.4%)	0.004
MPD diameter (mean ± SD) mm	6.24 ±1.17	5.14 ± 1.6	0.051
Intraductal calculi, n (%)	6 (40%)	19 (42.2%)	0.416
Dominant stricture, n (%)	4 (26.6%)	12 (26.6%)	0.545
CBD stricture, n (%)	1 (6.6%)	2 (4.4%)	0.384
Pseudocyst, n (%)	1 (6.6%)	2 (4.4%)	0.756
Steatorrhea, n (%) (prior to endotherapy)	2 (13.3%)	24 (53.3%)	0.007
HbA1c (post-endotherapy) g/dl	6.38 ± 1.64	8.07 ± 1.84	<0.001

## Discussion

CP is a condition characterized by inflammation and fibrosis with few treatment options. Even with excellent analgesia and nutrition, the patient’s quality of life is lacking. Although pain is a crucial factor contributing to the poor quality of life of these patients, complications of endocrine insufficiency also add to the burden. Our study demonstrated that 30% of patients with CP and endocrine dysfunction benefit from endoscopic ductal interventions.

The development of exocrine and/or endocrine insufficiency in patients with CP independently increases mortality and morbidity [12]. Endocrine insufficiency is associated with micro- as well as macroangiopathic complications, which adds to the disease burden. Delaying these complications by either medical or surgical means has not been well explored in studies. In our study, 31.9% of patients with endocrine dysfunction prior to endotherapy had improvement in their glycemic profile, as evidenced by their decreased requirement for insulin. Improvement in steatorrhea was seen in only 3.5% of patients. A recent retrospective study reported that the presence of MPD stricture and ductal calcification was independently associated with the development of PEI and diabetes mellitus [13]. Hence, clinical intuition would dictate that addressing these factors would lead to an improvement in these symptoms. However, the evidence to back this claim is heterogeneous. A multicenter study by Inui et al. showed improvement in exocrine and endocrine insufficiency in 38% and 24.3% of patients, respectively [14]. A study by Delhaye et al. followed up with patients with CP who had been treated endoscopically and demonstrated that the progression of both exocrine and endocrine insufficiency is slower in patients who have been treated with this modality [15]. A recent prospective follow-up study has demonstrated that pancreatic ductal calculi could increase the risk of developing diabetes in CP and that ductal clearance could delay the development of diabetes [16]. Earlier studies by Rösch et al. and Binmoeller et al. did not report any improvement in endocrine or exocrine insufficiency [17,18]. Similar results have been demonstrated by Adamek et al., Schneider et al., and Cahen et al. [19-21]. Lack of improvement in steatorrhea is explained as PEI develops in the late stage of the disease, and any intervention at this stage may not be helpful. This has been reflected in our study as well, as the majority of patients who did not show an improvement in endocrine dysfunction post-endotherapy had concomitant exocrine insufficiency.

Improvement in endocrine dysfunction in a subset of patients may point to the fact that intervening early and thereby preserving the tail of the pancreas and the islets of Langerhans may help in delaying or ameliorating diabetes in these patients. The mechanism postulated for this beneficial effect of endotherapy on glycemic status is a possible reduction in pancreatic stellate cell-mediated inflammation resulting from lowered ductal pressure [16]. In a significant proportion of patients who showed improvement, the etiology of pancreatitis was idiopathic (idiopathic chronic pancreatitis, ICP), and this was an independent predictor of glycemic status improvement. One explanation could be that patients with ICP have a milder clinical course with fewer CP-related complications and lower healthcare utilization as compared to patients with other etiologies [22]. However, in our study, improvement in glycemic parameters was observed in patients who had documented endocrine dysfunction prior to endotherapy. This result is interesting, as prior studies have concluded that there is no benefit of endotherapy in patients with already established diabetes [23].

Patient selection is key in order to obtain an optimal outcome with endotherapy. The morphological indications for endoscopic treatment have to be precise and restrictive. Decompression of the pancreatic duct post-endotherapy, leading to pain relief, lends credence to the fact that increased ductal pressure plays a dominant role in the pathophysiology of pain. Initial studies with pancreatic duct stenting both for stones as well as strictures report good short- and medium-term outcomes with respect to pain relief [18]. A trial of endotherapy can be judiciously used for assessing the response to pain with decompression prior to sending a patient for surgery.

Despite being one of the few studies that comprehensively looked at the question of the impact of endotherapy on the metabolic functions of the pancreas, our study does have many limitations. First, this is a retrospective single-center study, and hence selection bias is inherent. Second, being a single-center study, the experience may vary across different centers depending on the patient cohort and available endoscopic expertise. Third, we did not use ESWL as part of endotherapy as this was not available at our center. Fourth, we did not perform 75 g of OGTT for patients with endocrine dysfunction. This may have led to missing some early cases of glycemic alterations in patients with CP [24]. Fourth, the small sample size and limited follow-up duration limit the generalizability of our findings. Hence, adequately powered prospective studies are the need of the hour. Lastly, we did not find any independent predictive factor for improvement in endocrine dysfunction as we did not have statistically significant demographics to explain all the parameters.

## Conclusions

Pancreatic endotherapy has stood the test of time as a safe and effective noninvasive modality to treat pain in CP. The improvement in glycemic parameters in a significant proportion of these patients is promising. Further, large-scale, adequately powered prospective studies are needed to comprehensively answer the question of whether endotherapy is a viable option for treating endocrine insufficiency in these patients.
